# CA19-9 producing locally advanced papillary thyroid carcinoma: a case report

**DOI:** 10.1186/s40792-024-01887-w

**Published:** 2024-04-10

**Authors:** Hiroki Morikawa, Takaaki Oba, Ayaka Kitazawa, Ryoko Iji, Nami Kiyosawa, Masatsugu Amitani, Tadafumi Shimizu, Toshiharu Kanai, Takeshi Uehara, Ken-ichi Ito

**Affiliations:** 1 Division of Breast and Endocrine Surgery, Department of Surgery, Shinshu University School of Medicine, 3-1-1 Asahi, Matsumoto, Nagano Japan; 2 Division of Laboratory Medicine, Shinshu University Hospital, Matsumoto, Japan

**Keywords:** Papillary thyroid carcinoma, Tracheal invasion, CA19-9

## Abstract

**Background:**

CA19-9 is a tumor marker for gastrointestinal and biliary-pancreatic adenocarcinomas; however, its association with thyroid cancer is unknown. Here, we report a case of CA19-9 producing locally advanced papillary thyroid carcinoma (PTC).

**Case presentation:**

A 66-year-old woman who was identified with a thyroid tumor after a close examination of an elevated serum CA19-9 level, which was detected at health screening, was referred to our hospital. Ultrasonography revealed a 34 × 31 mm hypoechoic lesion in the lower pole of the left thyroid lobe. Computed tomography revealed a solid thyroid tumor with tracheal invasion without any distant metastases. Bronchoscopy revealed tumor exposure into the tracheal lumen on the left side of the trachea. Fine-needle aspiration cytology led to a diagnosis of papillary thyroid carcinoma (PTC). The patient underwent a total thyroidectomy, tracheal sleeve resection with end-to-end anastomosis, and lymph node dissection in the left cervical and superior mediastinal regions (D3c) with a reversed T-shaped upper sternotomy down to the third intercostal level. Histopathological analysis confirmed the diagnosis of PTC with tracheal invasion and no lymph node metastases (pT4a Ex2 N0). Immunohistochemical staining showed the expression of CA19-9 in cancer cells. Postoperatively, the serum CA19-9 level of the patient decreased to within the normal range.

**Conclusions:**

Some PTCs produce CA19-9, although less frequently. When elevated serum CA19-9 levels are observed, PTC should be included in the differential diagnosis for further investigation.

## Background

Carbohydrate antigen 19-9 (CA19-9) is a sensitive tumor marker for pancreatic and biliary malignancies [1]. However, its significance in thyroid cancer remains largely unexplored.

Papillary thyroid carcinoma (PTC) is the most prevalent histological type of thyroid cancer that originates from the thyroid follicular cells [2]. Thyroglobulin (Tg) serves as a tumor marker for PTC, particularly useful for monitoring recurrence in patients who have undergone total thyroidectomy. However, Tg is not a specific marker for PTC because serum Tg levels are elevated even in adenomatous goiters or follicular tumors [3, 4]. Despite this, Tg is the primary marker for monitoring PTC owing to the lack of other effective tumor markers. This makes it a commonly used indicator for tracking the progression of PTC.

Here, we report a case of locally advanced PTC with tracheal invasion which presents with elevated serum level of CA19-9. Immunohistochemical staining for the primary tumor showed the expression of CA19-9 on the cytoplasmic membrane of cancer cells. Following curative resection, the patient’s serum CA19-9 level decreased to the normal range, indicating that CA19-9 was produced by PTC cells.

## Case presentation

**Fig. 1:**
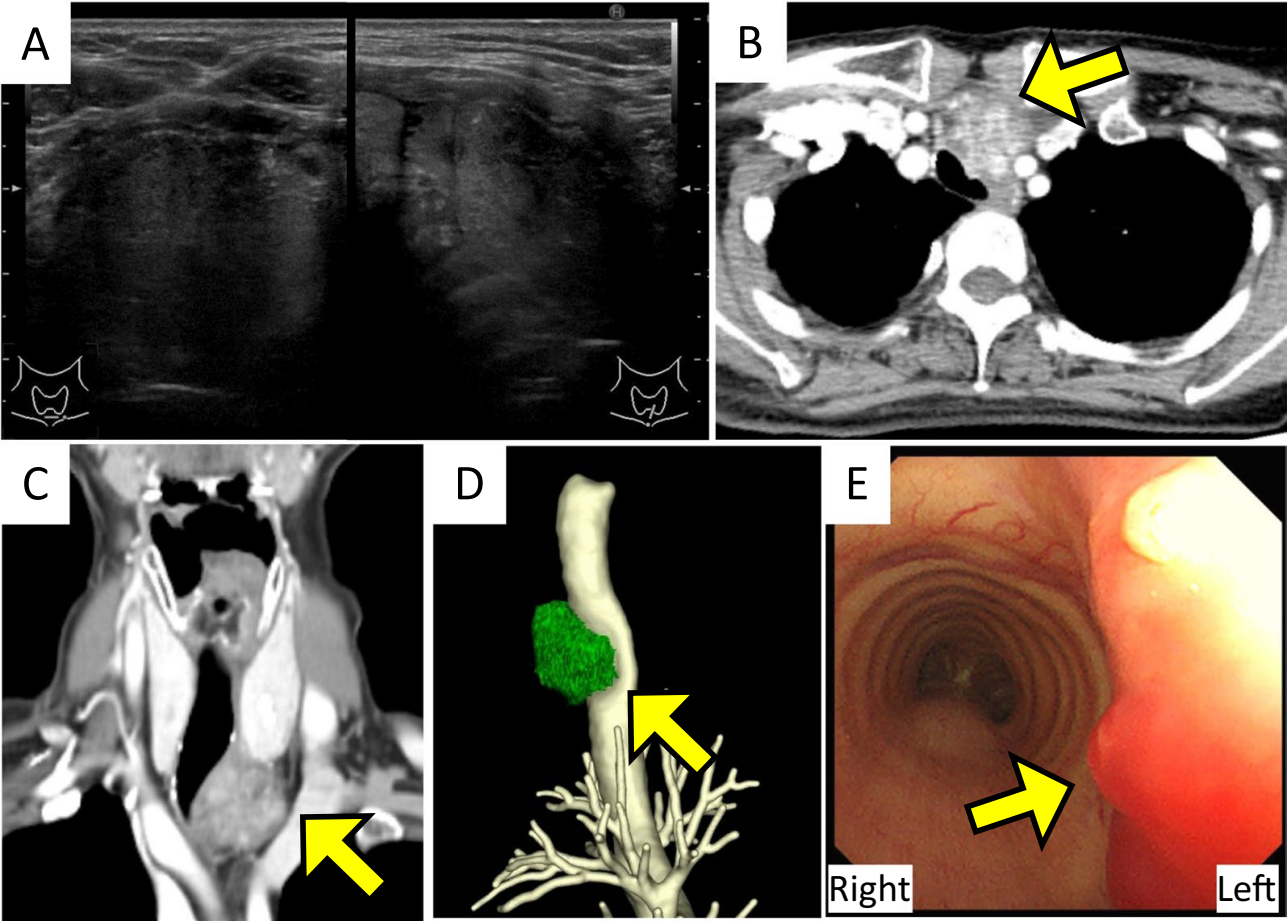
Imaging findings. **A** Ultrasonographic findings. A hypoechoic mass measuring 34 × 31 mm is observed in the lower pole of left thyroid lobe. **B**–**D** Computed tomography findings. A solid mass in the lower pole of left thyroid lobe (arrow) compresses the trachea and partially protrudes into the tracheal lumen, suggesting tracheal invasion. **B** Axial view, **C** coronal view, and **D** provides a 3D reconstructed view. **E** Bronchoscopic findings. A papillary structure approximately 3 cm in length (arrow) is visible within the tracheal lumen, indicative of tumor exposure

A 66-year-old woman with no history of malignant tumors was found to have elevated serum CA19-9 levels during a medical checkup. The patient underwent pancreatic and biliary examinations at a nearby primary hospital, which revealed no abnormal findings. Computed tomography (CT) revealed a tumor at the level of the suprasternal notch, which was diagnosed as PTC by fine-needle aspiration cytology. Subsequently, the patient was referred to our hospital for further examination and treatment. The serum CA19-9 level was elevated to 78.2 IU/mL (normal range, ≤ 37 IU/mL). Serum Tg level was within the normal range, and TgAb tested negative. Ultrasonography revealed a 34 × 31 mm hypoechoic nodule with an indistinct border extending caudally from the left lobe of the thyroid gland (Fig. 1A). CT revealed a heterogeneously enhanced mass extending from the lower pole of the left thyroid lobe to the posterior sternal region. The tumor significantly compressed the trachea and an irregular lesion was observed in the tracheal lumen, indicating that the tumor had invaded the entire tracheal wall (Fig. 1B–D). CT did not reveal any enlarged or suspicious metastatic lymph nodes from the neck to the mediastinum or any distant metastases. Bronchoscopy revealed an exposed tumor approximately 3 cm in length on the left side of the tracheal lumen (Fig. 1E).

**Fig. 2:**
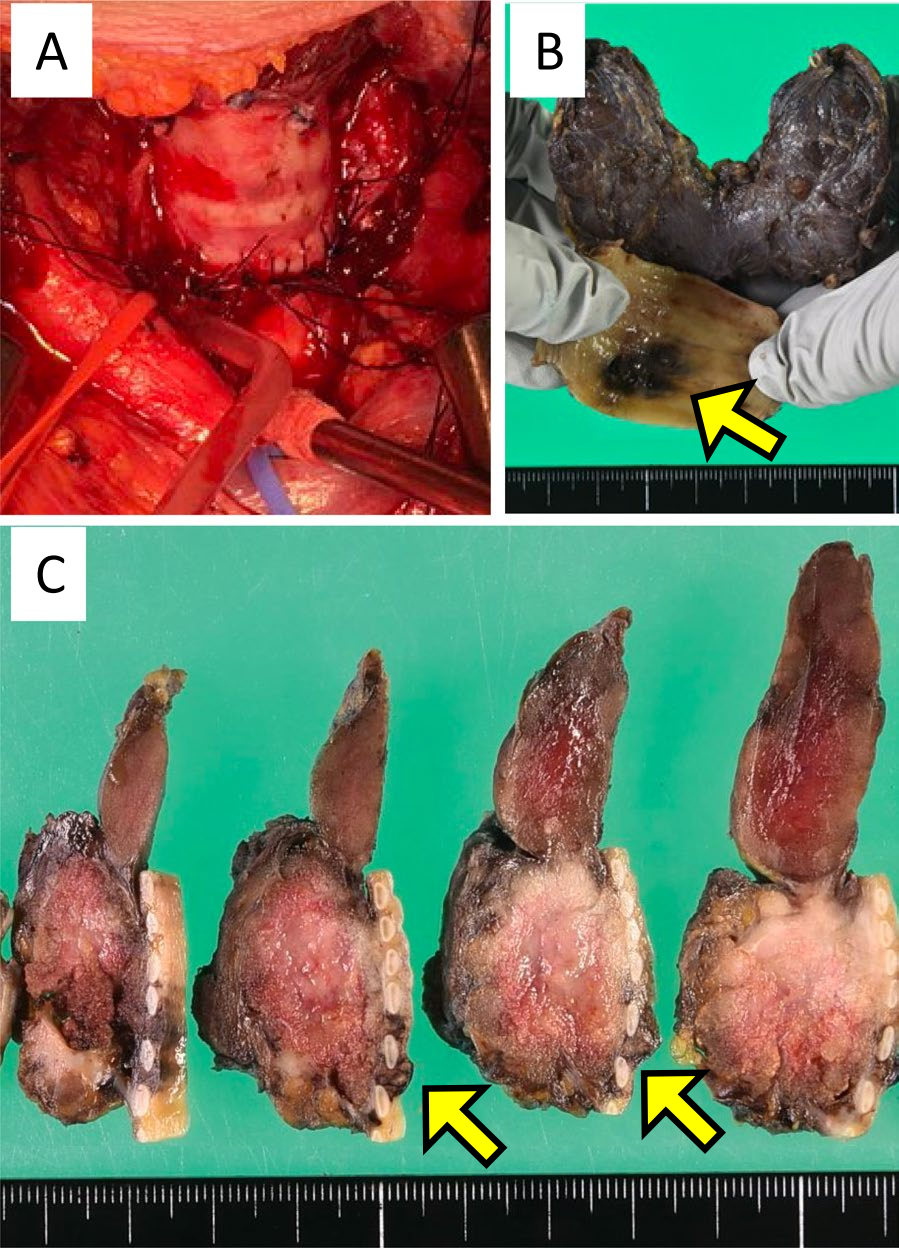
Intraoperative and gross findings of the resected specimens. **A** Intraoperative findings following total thyroidectomy and tracheal sleeve resection. Trachea was reconstructed with end-to-end anastomosis (arrow). **B**, **C** Gross findings of the resected specimens. The tumor is located in the lower pole of left thyroid lobe and demonstrates tracheal invasion (arrow)

The patient was diagnosed with PTC with tracheal invasion (cT4a Ex2 N0 M0, cStage III). Subsequently, the patient underwent total thyroidectomy, tracheal sleeve resection with concurrent end-to-end tracheal anastomosis, and left cervical and superior mediastinal node dissection (D3c) with a reversed T-shaped upper sternotomy down to the third intercostal level. Although the tumor adhered to the left recurrent laryngeal nerve, it was preserved without gross residual tumor tissue around it. Tracheal sleeve resection of 3.5 cm length, followed by end-to-end anastomosis of the trachea, was performed, and the anastomosis site was covered with thymic tissue (Fig. 2A). The absence of tumor exposure in both tracheal stumps using intraoperative rapid histopathological diagnosis was confirmed. In the resected specimen, the thyroid tumor invaded beyond the tracheal mucosa (Fig. 2B, C). The postoperative course was uneventful, and the patient was discharged 12 days postoperatively.

**Fig. 3:**
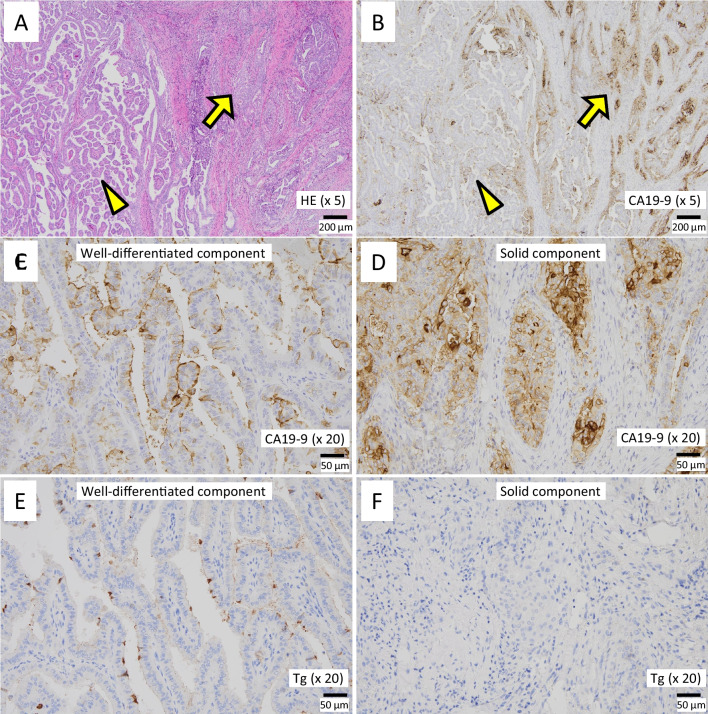
Histopathological findings. The majority of the tumor was composed of well-differentiated PTC (arrowhead in **A**; ×5), while a portion of the tumor showed a solid pattern (arrow in **A**; ×5). The expression level of CA19-9 (**C**, **D**; ×20) and thyroglobulin (Tg) (**E**, **F**; ×20) in the well-differentiated PTC section (**C**, **E**) and the portion of PTC with a solid pattern (**D**, **F**) are shown, respectively

**Fig. 4:**
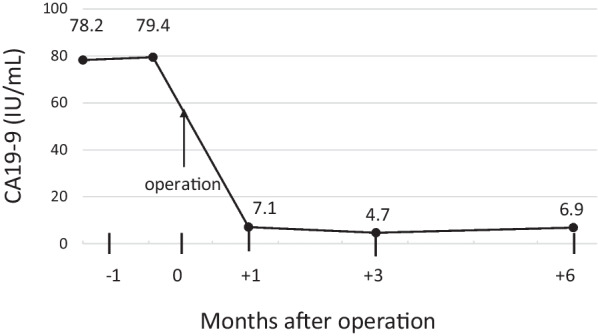
Time course of serum CA19-9 level. Post-surgical changes in serum CA19-9 levels. Serum CA19-9 levels decreased post-surgery, falling within normal limits

Histopathological examination of the excised specimen revealed PTC with tracheal invasion in the left thyroid lobe (pT4a and pEx2). While the majority of the tumor was composed of well-differentiated PTC, a portion of the tumor exhibited a solid pattern (Fig. 3A). In addition, intrathyroidal metastases measuring 7 and 4 mm in the left and right lobes, respectively, were detected. No lymph node metastasis (pN0) was observed. The histopathological stage was designated as pStage III. Immunohistochemical staining of the primary tumor demonstrated the expression of CA19-9 on cancer cells. The expression level of CA19-9 was higher in the solid pattern portion of the tumor than in the well-differentiated areas (Fig. 3B–D). Conversely, thyroglobulin levels were diminished in the solid pattern section of the tumor relative to the well-differentiated portion (Fig. 3E, F). The serum CA19-9 level decreased to the normal range (7.1 IU/mL) 1 month postoperatively (Fig. 4). Three months after the surgery, the patient received radioactive iodine (RAI) therapy (100 mCi). On 131-I scintigraphy following RAI therapy, the accumulation of 131-I was observed in the thyroid bed alone. However, 18 months had passed since the surgery, and the patient showed no signs of recurrence.

## Discussion

CA19-9 was first identified by Koprowski et al. in 1979; it reacted with a monoclonal antibody (1116 NS 19-9) that was synthesized by a hybridoma obtained from a mouse spleen inoculated with a human colorectal cancer cell line (SW1116) [5]. Subsequent research revealed its expression in normal epithelial cells of the gallbladder, biliary ducts, and pancreas [6]. CA19-9 is a carbohydrate antigen that accounts for a major proportion of the total molecular weight of mucin glycoproteins [6], which are abundantly distributed on the surface of epithelial cells and are associated with carcinogenesis and the malignant phenotype of cancer cells [7]. As its expression is increased by oncogenesis in the gallbladder, biliary ducts, and pancreas [6], CA19-9 is a useful marker for malignancies in these organs [1]. Moreover, its expression has been observed in various solid malignancies, such as hepatocellular carcinoma, gastric cancer, colorectal cancer, ovarian cancer, and endometrial cancer [8–11].

Recent reports indicate that medullary thyroid carcinoma (MTC), which originates from parafollicular cells, can produce CA19-9 [12]. Previous studies have underscored the prognostic significance of serum CA19-9 levels in patients with MTC [13–15]. In the context of PTC and CA19-9, Hashimoto et al. conducted a study with a cohort of 39 patients with PTC, in which 58% of the patients showed positive immunohistochemical staining for CA19-9 at diagnosis [16]. Similarly, Vierbuchen et al. documented the presence of CA19-9 immunostaining in PTC tissue samples, suggesting the potential for CA19-9 synthesis by PTC cells [17]. However, studies reporting serum CA19-9 levels in patients with PTC are sparse. Only two studies offering time course change in serum CA19-9 levels in patients with PTC were found through a comprehensive search of PubMed, employing the keywords “papillary thyroid carcinoma” and “CA19-9” [18, 19]. Kihara et al. described a case where liver metastasis from PTC was detected 16 year post-thyroidectomy, accompanied by elevated serum CA19-9 levels (326 IU/mL). Following partial liver resection, there was a significant decrease in serum CA19-9 concentrations (165 IU/mL) [18]. Moreover, Yamaguchi et al. reported a case with CA19-9 producing lung metastasis from PTC, which was diagnosed 15 years after total thyroidectomy for PTC. CA19-9 levels were elevated to 41.6 IU/m, but returned to the normal range, at 28.4 IU/mL, after the resection of pulmonary metastases [19]. Furthermore, in the two cases, CA19-9 immunostaining of the metastatic sites revealed CA19-9 localization within the cytoplasm of cancer cells. In contrast to these previous two cases, our patient showed elevated serum CA19-9 levels at diagnosis, but not when distant metastases were observed. Immunohistochemical staining of the primary tumor of our patient revealed CA19-9 in the cytoplasmic membrane of cancer cells, suggesting that elevated serum CA19-9 levels were due to leakage from PTC cells into the bloodstream. The hypothesis is supported by the subsequent normalization of serum CA19-9 levels following curative resection.

In the present case of PTC, elevated serum CA19-9 levels were observed. Among other carbohydrate antigens used as tumor markers, increased expression of Carbohydrate Antigen 15-3 (CA 15-3), a recognized tumor marker for breast cancer [20], which is associated with tumor aggressiveness [21, 22], has been reported in subsets of PTC. The expression of these carbohydrate antigens is associated with the differentiation status of cancers [23–25]. Our previous study demonstrated that the expression levels of polypeptide *N*-acetylgalactosaminyl transferase-3 (GalNAc-T3), a pivotal enzyme in carbohydrate antigen biosynthesis, decreased concurrently with the de-differentiation process in thyroid cancer [26]. This suggests that the proteins modulating carbohydrate antigen expression change during de-differentiation in thyroid cancer. This affects the expression of carbohydrate antigens in cancer cells derived from the thyroid follicular epithelial or parafollicular cells.

In accordance with this, it has been reported that a few PTC cells may begin producing CA19-9 as they acquire an aggressive phenotype or metastatic potential. In a report by Yamaguchi et al. [19], primary PTC cells in the thyroid gland revealed no CA19-9 production, despite the presence of CA19-9 in pulmonary metastatic lesions. Similarly, Ogawa et al. documented a case of anaplastic thyroid cancer (ATC) exhibiting elevated serum CA19-9 levels during anaplastic transformation from PTC [27]. Consistent with these findings, in our case, PTC cells with a solid pattern, indicative of a poorly differentiated component, demonstrated higher CA19-9 expression compared to the well-differentiated sections of PTC. Furthermore, the expression level of thyroglobulin, which is a differentiation marker of thyroid cancer [26], was lower in the solid component of the tumor than in the well-differentiated areas. This inverse staining pattern between CA19-9 and thyroglobulin may support the notion that some PTC cells can produce CA19-9 as they evolve into a more malignant phenotype or undergo a de-differentiation process.

## Conclusion

Some PTCs produce CA19-9 while acquiring malignant phenotypes. Therefore, when elevated serum CA19-9 levels are observed, PTC should be included in the differential diagnosis for further investigation.

## Data Availability

The datasets used in this case report are available from the corresponding author on reasonable request.

## Abbreviations

CA19-9: Carbohydrate antigen 19-9
PTC: Papillary thyroid carcinoma
CT: Computed tomography
FNAC: Fine needle aspiration cytology
Tg: Thyroglobulin
Tg Ab: Thyroglobulin antibody
MRI: Magnetic resonance imaging
MTC: Medullary thyroid carcinoma
CEA: Carcinoembryonic antigen
ATC: Anaplastic thyroid carcinoma
